# Complete genome sequence of Japanese RSSC phylotype-I strains infecting different host plants

**DOI:** 10.1128/mra.00483-24

**Published:** 2024-06-25

**Authors:** Lokendra Rana, Miho Satoh, Masayuki Tsuzuki, Akinori Kiba, Yasufumi Hikichi, Kouhei Ohnishi

**Affiliations:** 1 The United Graduate School of Agricultural Sciences, Ehime University, Matsuyama, Ehime, Japan; 2 Institute of Molecular Genetics, Kochi University, Nankoku, Kochi, Japan; 3 Faculty of Agriculture and Marine Science, Kochi University, Nankoku, Kochi, Japan; DOE Joint Genome Institute, Berkeley, California, USA

**Keywords:** type III effector, RSSC, tomato, potato

## Abstract

*Ralstonia solanacearum* species complex (RSSC) shows a broad host range and is classified into four phylotypes. To compare type III effectors, we have determined the complete genome sequences of several RSSC strains, especially phylotype-I strains isolated in Japan, with different host specificity.

## ANNOUNCEMENT

*Ralstonia solanacearum* species complex (RSSC), a Gram-negative bacterium within the *Burkholderiales*, infects over 200 plant species and is classified into four phylotypes (1). RSSC is differentiated into three distinct species: *Ralstonia pseudosolanacearum* (phylotypes I and III), *R. solanacearum* (phylotype IIA and IIB), and *Ralstonia syzygii* (phylotype IV) (2). Most phylotype-I and some phylotype-IV strains have been isolated in Japan (3). While Japanese phylotype-IV strains infect only potato species (*Solanum tuberosum*), phylotype-I strains show a broad host range. Repertoires of type III effectors (T3Es) mainly determine the RSSC host range. We determined the complete genome sequences of five phylotype-I strains and one phylotype-IV strain to investigate RSSC host specificity. The six strains isolated from different plants (Table 1) were obtained as freeze-dried cultures from the Research Center of Genetic Resources, NARO (Tsukuba, Japan). Phylotypes determined by the research center using the nucleotide sequences of 16S rDNA and *egl* gene are listed in Table 1.

**TABLE 1:** Characterization of six RSSC strains

Strain	Isolated from, where, and when	Phylotype 16S rDNA *egl*	BioProject BioSample	Sequencing	DRAaccession no.	Number of reads	*N*_50_ value (bp)		Accession no.	Genome size (bp)	GC content (%)	Coverage (×)	Number of genes
MAFF 211519	Eggplant whole plant, Saga, 1986	I	PRJDB17010	MiSeq	DRX496971	6,577,441				5,746,956	66.9	164	
		16 S	SAMD00657122	MinION	DRX496977	27,369	13,751	Chromosome	AP029004	3,791,125	66.8		3,506
		egl						Megaplasmid	AP029005	1,955,831	67.3		1,525
MAFF 211520	Eggplant whole plant, Saga, 1986	I	PRJDB17010	MiSeq	DRX496972	4,769,180				5,953,698	66.8	219	
		16 S	SAMD00657123	MinION	DRX496978	110,585	13,960	Chromosome	AP029006	3,977,427	66.6		3,692
		egl						Megaplasmid	AP029007	1,976,271	67.2		1,536
MAFF 211521	Potato whole plant, Saga, 1986	I	PRJDB17010	MiSeq	DRX496973	7,899,021				5,953,694	66.8	186	
		16 S	SAMD00657124	MinION	DRX496979	23,363	7,706	Chromosome	AP029008	3,977,428	66.6		3,703
		egl						Megaplasmid	AP029009	1,976,266	67.2		1,541
MAFF 301524	Potato whole plant, Nara, 1982	I	PRJDB17010	MiSeq	DRX496974	2,466,258				5,952,515	66.8	129	
		16 S	SAMD00657125	MinION	DRX496980	51,101	23,583	Chromosome	AP029010	3,892,407	66.7		3,608
		egl						Megaplasmid	AP029011	2,060,108	66.9		1,649
MAFF 301069	Tobacco whole plant, Shizuoka, 1965	I	PRJDB17010	MiSeq	DRX496975	1,547,700				5,746,698	66.9	89	
		16 S	SAMD00657126	MinION	DRX496981	39,092	12,907	Chromosome	AP029012	3,741,881	66.9		3,424
		egl						Megaplasmid	AP029013	2,004,817	67.0		1,583
MAFF 211271	Potato stem, Shizuoka, 1994	IV	PRJDB17010	MiSeq	DRX496976	2,765,254				5,521,407	66.4	194	
		16 S	SAMD00657127	MinION	DRX496982	30,834	19,249	Chromosome	AP029014	3,532,797	66.4		3,199
		egl						Megaplasmid	AP029015	1,988,610	66.4		1,558

Strains were streaked on B media (1% bactopeptone, 0.1% yeast extract, and 0.1% casamino acids) with 1.5% agar. Genomic DNA was extracted from cells grown overnight in B media at 28°C using a Wizard HMW DNA Extraction Kit (Promega). The purified DNA was used for both short-read and long-read sequencing. For short-read sequencing, genomic DNA was fragmented to 150–350 bp, and libraries were built using the NEBNext Ultra II FS DNA Library Prep Kit for Illumina (NEB) with NEBNext Multiplex Oligos for Illumina (NEB). The pooled libraries (at a concentration of 20 pM) were sequenced on an Illumina MiSeq (MiSeq Reagent Kit v3, 150 cycles), generating 75 bp paired-end reads. Reads with Phred-encoded quality scores of more than 30 were selected using MiSeq Reporter 2.6.2. For long-read sequencing, genomic DNA was processed with a 1D Ligation Sequencing kit (SQK-LSK109, Oxford Nanopore Technologies) with Native Barcoding Expansion (EXP-NBD104). The pooled libraries were sequenced without size selection on MinION with Flow Cell (R9.4.1) under the control of MinKNOW version 22.05.5 with a fast model of basecalling. The quality score was measured on the logarithmic Phred scale. Reads below the minimum quality score of 8 using MinKNOW version 22.05.5 were removed as failed reads. The hybrid assembly with long and short reads was conducted using an assembly pipeline for bacterial genomes, Trycycler version 0.5.4 (4). Trycycler uses TBLASTN to search for the *dnaA* allele in each completed replicon, and the sequence was circularized (5). Gene annotation was conducted using DFAST prokaryotic genome annotation pipeline version 1.6.0 (6). Default parameters were used for all software unless otherwise specified.

Characteristics of the sequenced genomes are listed in Table 1. The complete genome sequences of six RSSC strains indicated that all strains have two replicons, a chromosome, and a megaplasmid, like other RSSC strains (7). GC contents of all strains were over 66%, which is consistent with other RSSC strains (8). Bacterial T3Es were scanned at Ralsto T3E (9) (https://iant.toulouse.inra.fr/bacteria/annotation/site/prj/T3Ev3/) on 14 February 2024, and more than 70 T3Es were detected in all strains.

## Data Availability

This whole-genome shotgun (WGS) project has been deposited at DDBJ/EMBL/GenBank. The versions described in this paper are the first versions. The read archives have been deposited in DDBJ DRA. The genome nucleotide and DRA accession numbers are listed in Table 1.
